# Cryopreserved Placental Membrane Allograft Reduces the Likelihood of Developing a New or Recurring Foot Ulcer and All-Cause Mortality in Diabetic Patients When Compared with Other Cellular- and Tissue-Based Products

**DOI:** 10.1089/wound.2021.0123

**Published:** 2023-01-18

**Authors:** Joan DaVanzo, Alex Hartzman, Christopher Surfield, Allen Dobson

**Affiliations:** Dobson DaVanzo and Associates, LLC, Vienna, Virginia, USA.

**Keywords:** diabetic foot ulcer, DFU, Cellular- and Tissue-Based Products, CTPs, Medicare

## Abstract

**Objective:**

To compare outcomes for Medicare patients with diabetic foot ulcer(s) (DFU) receiving cryopreserved placental membrane containing viable cells (vCPM) to other Cellular- and Tissue-Based Products (CTPs).

**Approach:**

Patients with DFU and CTP use were selected in Medicare claims (2013–2017) by using a strict definition of DFU with demonstrated diabetes etiology. We compared the effectiveness of vCPM with other CTPs on: (1) reduction of post-treatment ulcer occurrence, and (2) reduction in 1 year mortality. We controlled for selection bias and differential risk characteristics between comparison groups in a two-stage inverse probability treatment weighting model.

**Results:**

Overall, 7,869 DFU episodes with CTP use met inclusion criteria: 786 received vCPM, 4,546 received another “cellular” CTP, and 2,537 received “acellular” CTP. For ulcer occurrence, we examined: 30-, 90-, 180-, and 365 days post-treatment. We found a significant reduction in ulcers at each period for vCPM compared with either alternative CTP—results range from a 36.7% percentage point reduction in ulcer occurrence at 30 days compared with cellular CTP, and a 58.5% percentage point reduction at 365 days compared with acellular CTP. Further, the application of vCPM reduces mortality within 1 year by 2.3 percentage points (13–13.8% change) compared with other CTPs.

**Innovation:**

This study examines the differences in ulcer occurrence and mortality for Medicare DFU patients receiving vCPM and other CTPs. Our strict DFU definition excludes beneficiaries without foot ulcer with demonstrated diabetes etiology.

**Conclusion:**

Among CTPs, vCPM users have reduced ulcer rates (recurrent or new) and reduced all-cause mortality compared with other “cellular” and “acellular” CTPs.

**Figure:**
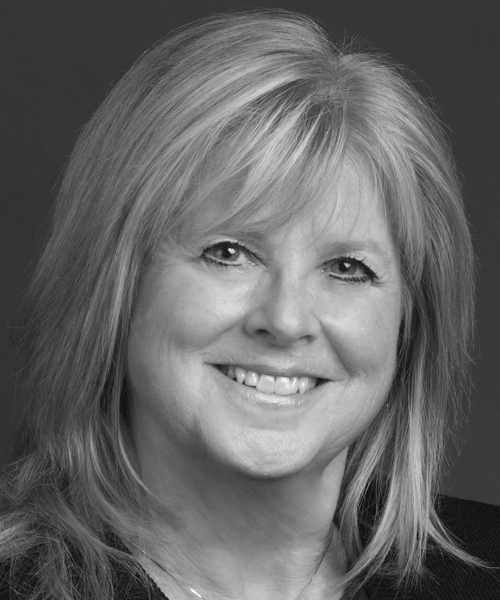
Joan DaVanzo, PhD, MSW

## Introduction

Diabetic foot ulcers (DFUs) can be debilitating wounds that form in 15–25% patients with diabetes and, if not properly managed, can lead to infection, amputation, and, possibly, death.^1,2^ In the Medicare Fee for Service (FFS) program, a wound is considered chronic and eligible for advanced wound care protocols if it has not closed within at least 4 weeks after initial diagnosis.^3^ Relatively recent advances in wound care technology have improved outcomes among patients with DFU.

Cellular- and Tissue- Products (CTPs) are a relatively new technological component of advanced wound care. This study presents the outcomes of using CTPs (cryopreserved placental membrane containing viable cells [vCPM]) compared with other cellular CTPs and acellular CTPs, examining the differences in post-treatment ulcer occurrence and mortality for DFU patients treated in the Medicare outpatient hospital setting.

vCPM or “cryopreserved placental membrane with viable cells” is an FDA-regulation of Human Cells, Tissues, and Cellular and Tissue-Based Products (HCT/Ps) allograft derived from the human placental membrane and processed in such a way that maintains the integrity of the extracellular matrix (ECM) and growth factors and the viability of the native cells of the placenta, including mesenchymal stem cells. This composition is unique to the vCPM and other technologies may lack one or more of these components.

*In vitro* studies have shown that the ECM provides three-dimensional structural support as well as a physiological microenvironment for cells and proteins that promotes cellular adhesion and migration, in addition to protecting growth factor functionality that can influence different stages of wound healing. Other *in vitro* and *in vivo* studies have shown that mesenchymal stem cells (MSCs) provide matrix proteins, cytokines, and growth factors that coordinate the tissue repair process through downregulation of inflammation, stimulation of blood vessel formation, and recruitment and support of fibroblasts and epithelial cells for rapid wound closure.^4^

### Clinical Problems Addressed

Multiple classes of CTPs are available for the treatment of chronic DFUs. The CTPs are an advanced treatment (AT) and are considered an improvement over standard of care for chronic wounds that typically consists of debridement, cleaning, application of dressings, treating related infections, restoring blood flow to the wound site, and offloading.^5^ When these methods do not close wounds within 4 weeks, advanced care treatments may be beneficial.

The CTPs vary primarily in the composition, tissue source, and preservation methods, and they can be grouped into two major categories of cellular CTPs (containing living cells, either native to the tissue or exogenous), of which vCPM is included, and acellular (tissue-derived but without living cells). (Synthetic products are also available, and though acellular, they are not included in this analysis.) Individual branded CTPs vary in how they are preserved, packaged, and stored, which affects product composition, ease-of-use, and clinical effectiveness.^6^

Human placental membranes are a rich source of different cell types such as fibroblasts, epithelial cells, and stem cells along with collagen matrix and growth factors. Since different cells have different paracrine secretions, this may provide a benefit over a bioengineered CTP with only one cell type.

Currently, there are many commercial placental products, but they are preserved in different ways. The first type is when the tissue is devitalized/dehydrated, which means they have the ECM and some growth factors but no living cells. The second category is cryopreserved to better preserve the growth factors, but the process is not optimized to retain cell viability. vCPM is the third type where the processing retains viability of the native cells of the placenta, along with the ECM and growth factors and is, therefore, closer to fresh placental tissue.

*In vitro* studies demonstrate that the preservation of endogenous viable cells in placental membranes enhances the anti-inflammatory, antioxidant, chemo-attractive, and angiogenic activities of placental membranes compared with devitalized membranes.^7–9^

In clinical studies, treatment with vCPM has been shown to lead to higher wound closure rates as compared with devitalized placental tissue or bioengineered CTP with one cell type.

This study sought to compare the effectiveness of vCPM with other CTPs on reduction of post-episode ulcer occurrence, and mortality, utilizing secondary data from Medicare patients receiving treatment in real-world hospital outpatient settings.

## Materials

Medicare Fee-For-Service claims found in the Centers for Medicare & Medicaid Services (CMS) 100% Limited Data Set (LDS) were used in this study. Specifically, LDS institutional files (outpatient hospital, inpatient hospital, skilled nursing facilities, home health agencies, and hospice) from January 2012 to December 2018 were used to identify patients' co-morbidities and treatments. Medicare beneficiaries included in this analysis were continuously enrolled in Medicare Part A and B but were not enrolled in Part C (Medicare Advantage).

These data were obtained via a data use agreement with CMS with an Institutional Review Board (IRB) exemption, as no human subject participation was required. The outcomes of interest are: (1) the occurrence of ulcers (recurrent or new) after the end of the CTP application episode, (2) postepisode mortality, and (3) other related infections.

## Methods

After applying a stringent, claims-based chronic DFU definition, we constructed measures of patient risk and service use during the advanced wound care episode, and postepisode outcomes. The exclusions were designed to avoid confounding DFUs with other ulcers of the lower extremities.^10–12^

One challenge in creating episodes of care is having a “clean” period in which the patient does not have a DFU, and he or she has a full year of Medicare enrollment after treatment with a CTP (allowing for mortality outcomes). Ultimately, we applied a two-stage matching model for this cross-sectional study design. The first stage matching model addressed selection bias, whereas the second model was used to estimate the effect of CTP choice on post-treatment ulcers and mortality. Table 1 contains our inclusion/exclusion criteria for the sample of Medicare beneficiaries.

**Table 1.** Inclusion/exclusion criteria for study sample (Medicare beneficiaries)

Data: 100% Medicare Limited Dataset Institutional Files (Hospital Inpatient, Outpatient, Skilled Nursing Facility, Home Health, Hospice) 2012–2018		
		*n* = 15,625,783
Beneficiaries with diabetes (at least two diagnoses)	ICD-9-CM 249.xx or 250.xx, ICD-10-CM E08.xx -E13. xx	*n* = 12,362,182
ID lower limb chronic ulcers, potential DFU	ICD-9-CM codes 707.1x or ICD-10-CM code L97	*n* = 1,365,609
ID DFU beneficiaries	ICD-9-CM 707.14 or 707.15, ICD-10-CM L97.4- or L97.5	*n* = 935,438
Include only patients with: diabetic etiology, and principal and secondary diagnoses diabetes and lower limb ulcer	**I**CD-10-CM Exx.621 or Exx.622	*n* = 766,318
DFU patients with at least one outpatient hospital claim	Claim place of service code 22	*n* = 508,693
Episode construction ≤90 days		
No other ulcers of the nonlower limbs on the same claim, DFU at least 4 weeks, only paid CTP applications	Gap of 60 days between applications, Medicare coverage in pre- and post- period	*n* = 7,869

CTP, Cellular- and Tissue- Products; DFU, diabetic foot ulcer.

**FIGURE 1.**
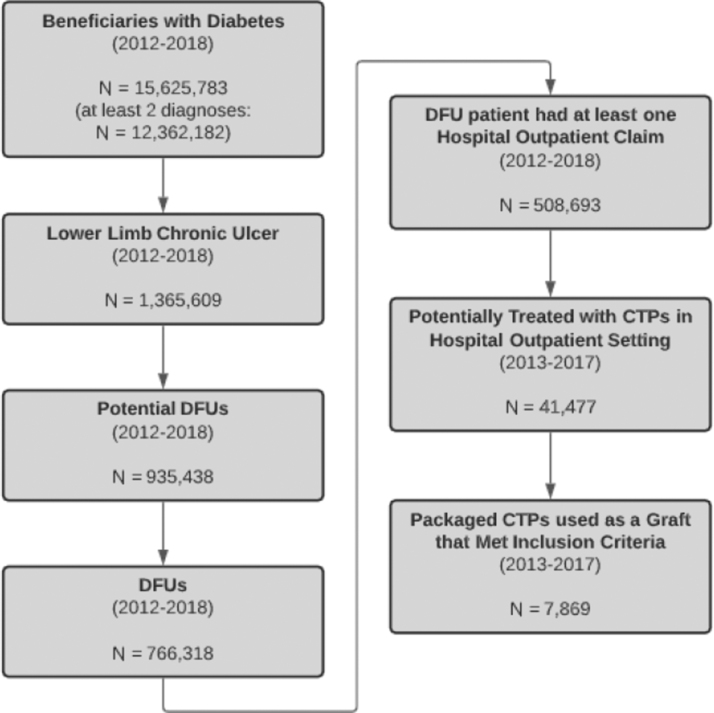
Study CONSORT diagram describing study inclusion and exclusion criteria. Study selection began with Medicare enrolled beneficiaries with diabetes, eventually reduced to enrollees who received a study-eligible CTP for a claims-confirmed diabetic foot ulcer. CTP, Cellular- and Tissue-Based Products; DFU, diabetic foot ulcer.

The identification of DFUs from Medicare claims data involved three main stages, which altogether ensured that the study sample was restricted to patients who had a foot ulcer with diabetic wound etiology. The CONSORT diagram (Fig. 1) shows the steps; the sample size resulting after exclusions was applied. In the first step, patients with at least one outpatient claim with a diabetes diagnosis during the years 2012–2018 were selected from the LDS.

The International Classification of Diseases, Ninth and Tenth Revision, Clinical Modification (ICD-9-CM and ICD-10-CM) codes were used to identify these patients, whose subsequent selection criteria are shown next in Fig. 1. Further, we assumed that all applications of CTP were applied to the same ulcer if the gap between two paid applications was no longer than 60 days. Finally, we limited clinical episodes of CTP application to 90 days as longer episodes may exceed the Medicare advanced wound care benefit.

### Statistical approach

First, we wanted to control for selection bias in that some beneficiaries might be more or less likely to receive vCPM. A stepwise logit model was used to identify which characteristics predicted the use of vCPM CTP for advanced wound care compared with either other cellular CTPs or acellular CTPs. Those variables with a coefficient estimate significant at the *p* < 0.20 level were included in the subsequent inverse probability treatment weight (IPTW) matching models.

Propensity score methods are statistical techniques that researchers use in observational studies for removing the effects of selection bias and confounding factors when estimating the treatment effects of an intervention or a policy change. This method is based on the propensity score, that is, the probability of treatment assignment conditional on observed baseline covariates. It is sometimes referred to as a “balancing score,” meaning that subjects who have the same propensity score regardless of their treatment status (*i.e.*, study group participants vs. comparison group members) also have the same distribution of their baseline characteristics.

The use of IPTW is similar to survey sampling weights that researchers use to adjust survey samples so that they are representative of specific populations. Joffe *et al.*^13^ describe how regression models can be combined with weighting by the inverse probability of treatment to calculate causal treatment effects. This is an important factor in addressing self-selection from the perspective of treatment effect estimation. Rubin later argues that an advantage of the use of propensity score methods is that they allow observational studies to be designed to approximate the results of randomized experiments.^14^

To account for sample size differences between test and comparison groups, we applied available data on beneficiary characteristics, risk, and wound characteristics to predict which group a beneficiary would be in using a stepwise logit regression.

Table 2 contains the variables used in matching vCPM to other cellular products and acellular products (our two comparison groups). We also matched on risk factors related to overall health (count of Condition Classification Software [CCS] conditions) and those related directly to DFU (such as gangrene, osteomyelitis, foot and toe cellulitis or abscess, paronchia). Other episode characteristics were number of institutional days, number of surgical debridements, number of non surgical debridements, and number of dehiscences.

**Table 2.** Control variables included in inverse probability weight model

Variable	Cellular	Acellular
	Comparison	Comparison
CTP area	^*^	^*^
Count of CCS conditions	^*^	^*^
Count of DFU risk conditions	^*^	^*^
Race (Other)	^*^	^*^
Race (Black)		^*^
Chronic kidney and renal disease	^*^	
Coronary disease	^*^	^*^
Nephropathy	^*^	^*^
Lung cancer	^*^	^*^
Osteomyelitis		^*^
Phlebitis/thrombophlebitis		^*^
Septicemia		^*^
Wheelchair disability		^*^

CCS Conditions, Condition Classification Software.

The IPTW model is a two-stage model in that it first estimates the probability of a patient receiving vCPM CTP relative to a counterpart, and it then uses these estimated probabilities as weights in the subsequent regression model to estimate the effect of vCPM. The propensity score is used to create a synthetic sample in which the distribution of measured baseline covariates is independent of study group assignment. One key advantage of using the IPTW matching model over other types of matching models is that all observations are able to be matched, thus allowing them to be retained.

This provides us with a richer, and more complete, sample for the second-stage estimation. With only propensity score matching, sometimes there are no comparison group members to match with study group members, which reduces the sample size. Variables used in matching are summarized next in Table 2, and risk factors related to overall health (count of CCS conditions) and those related directly to DFU.^15^

## Results

After the application of inclusion and exclusion criteria, a total of 7,869 Medicare beneficiaries with a DFU who were treated for 90 days with a CTP in an outpatient hospital setting between 2013 and 2017 were identified. Of these identified cases, 786 (10%) had vCPM CTP applied, 4,546 (58%) had a different cellular CTP applied, and 2,537 (32%) had an acellular CTP applied. Table 3 summarizes the results for vCPM compared with both other cellular and acellular CTPs.

**Table 3.** vCPM comparative performance on ulcer diagnosis post-CTP application episode

Comparison	Measure	30 Days	90 Days	180 Days	365 Days
	Average of vCPM	0.454	0.754	0.996	1.161
Other cellular	Average of other cellular products	0.717	1.285	1.748	2.152
	Estimated vCPM mean differential	−0.263	−0.531	−0.752	−0.991
	*p*-Value of mean differential	<0.001	<0.001	<0.001	<0.001
	95% CI of mean differential	−0.375 to −0.151	−0.722 to −0.341	−1.00 to −0.499	−1.286 to −0.697
	Standard error of mean differential	−0.057	−0.097	−0.129	−0.150
	Percentage point difference	−36.7%	−41.3%	−43.0%	−46.1%
Acellular	Average of acellular products	0.810	1.465	2.170	2.797
	Estimated vCPM mean differential	−0.356	−0.712	−1.174	−1.637
	*p*-Value of mean differential	0.001	<0.001	0.002	<0.001
	95% CI of mean differential	−0.563 to −0.148	−1.092 to −0.332	−1.935 to −0.413	−2.463 to −0.810
	Standard error of mean differential	−0.106	−0.194	−0.388	−0.422
	Percentage point difference	−44.0%	−48.6%	−54.1%	−58.5%

vCPM, cryopreserved placental membrane with viable cells.

Table 3 shows the likelihood of a beneficiary having a claim with an ulcer diagnosis at 30-, 90-, 180-, and 365 days after completing treatment with the CTP. There is a significant reduction in ulcers at each time period for vCPM compared with either alternative CTP group. For example, results in the first column suggest that 45% of patients receiving vCPM developed an ulcer within 30 days of treatment, compared with 72% of those receiving other cellular treatments. This points to a 26% reduction (and a 36.7 percentage point reduction), which is significantly different from 0 at the 0.001 level. Results range from a 36.7% to 58.5% percentage point reduction in postepisode ulcer occurrence.

Table 4 below shows the mortality outcomes at 365 days (1 year) after the end of treatment with the CTP for vCPM compared with both other cellular and acellular CTPs. After other episode characteristics are taken into consideration, the application of vCPM to a DFU indirectly results in an average mortality rate of 12.3%. Treatment with another cellular CTP results in an average mortality rate of 17.7%, and treatment with an acellular CTP results in an average mortality rate of 18.1%. vCPM cannot save lives but may have an indirect effect on mortality, possibly by way of closing wounds faster as an open chronic wound may ultimately lead to reduced Quality of Life (QoL), amputations *etc.*

**Table 4.** vCPM comparative performance on mortality 365 days after CTP application episode

Comparison	Measure	365 Day Mortality
	Average of vCPM (%)	0.123
Other cellular	Average of other cellular products (%)	0.177
	Estimated vCPM mean differential	−0.794
	*p*-Value of mean differential	0.010
	95% CI of mean differential	−0.396 to −0.192
	Standard error of mean differential	−0.307
	Marginal effect	[−0.023]
Acellular	Average of acellular products (%)	0.181
	Estimated vCPM mean differential	−0.869
	*p*-Value of mean differential	0.012
	95% CI of mean differential	−1.547 to −0.191
	Standard error of mean differential	−0.346
	Marginal effect	[−0.025]

Postepisode occurrence of cellulitis/abscess of foot or toe, gangrene, osteomyelitis, and paronychia was also tested. vCPM use relative to other cellular CTPs indicated a lower occurrence of cellulitis/abscess of the foot at 30- (*p* = 0.012), 90-, 180-, and 365 days (*p* < 0.001); cellulitis/abscess of the toe at 90- (*p* = 0.084), 180- (*p* = 0.045), and 365 days (*p* = 0.030); and osteomyelitis at 365 days (*p* = 0.051). Compared with acellular CTPs, vCPM indicated improvement on cellulitis/abscess of the foot at 90 days (*p* = 0.080) and osteomyelitis at 180- (*p* = 0.094) and 365 days (*p* = 0.076). Postapplication occurrence of gangrene and paronychia infections were tested, and we found a mix of results indicating nonsignificant difference relative to other cellular and acellular CTPs.

## Discussion

This claims-based observational study compared advanced CTPs as applied in the Medicare chronic DFU population treated in an outpatient hospital setting. We applied a rigorous process to identify DFUs from Medicare claims data and ensured that the study sample was restricted to patients treated with a CTP who had a foot ulcer with diabetic wound etiology. For example, we selected patients with a diagnosis of diabetes that was before the claim with the ulcer.

Among them, we only kept patients with at least two diagnoses of diabetes on their claims. In creating episodes of care, we had a “clean” period in which the patient did not have a DFU. We distinguished between lower extremity ulcers and only retained patients with the ulcer on their foot as indicated through the ICD-10 code. We only retained cases if the Healthcare Common Procedure Coding System (HCPCS) code for CTP and the DFU diagnostic codes were on the same claim.

Other studies have not been as restrictive. For example, Holzer *et al.* identified DFU patients from inpatient and outpatient claims data. Any patient with one or more claims containing a foot ulcer-related diagnosis or procedure in any fields was identified as having the DFU diagnosis.^16^ Tesfamichael and colleagues used a trauma diagnosis to exclude non-DFU patients. Diabetic patients who had a traumatic ulcer due to a car accident and those diabetic patients who were severely ill and unable to communicate throughout the study period were excluded. Those diabetic patients who had any diabetic-related ulcer were included in their study.^17^

We used Medicare 100% FFS hospital outpatient claims data from 2012 to 2018 to build the episodes in our study. These data are generated by providers who are billing for treating their Medicare patients in an advanced therapy episode for a DFU. Because the Medicare claims are used for payment, they contain information relevant to payment and very little clinical information. The service is indicated with a CTP or HCPCS code, date of service, site of service, allowed amount (the amount Medicare allows for that procedure), and Medicare paid amount.

Although a recent multicenter randomized clinical trial (RCT) compares vCPM with a cellular product, it does not answer the bigger question of how vCPM compares with other cellular and acellular CTPs.^18^ We, therefore, hope that the data in this article add to the information we have learned from this RCT. We also considered a comparative outcomes analysis evaluating clinical effectiveness in two different human placental membrane products for wound management that compares vCPM with an acellular allograft.^19^

We did not compare with usual care, as we wanted the groups to be equivalent in receipt of a CTP. Controlling for available case risk and selection bias, vCPM demonstrated both a greater reduction in the likelihood of having a postepisode ulcer and a reduction in the likelihood of death. In this population, post-CTP application episode foot or toe amputations were too rare in the final sample to estimate multivariate regression models. Differences in CTP performance are biologically plausible due to differences in material make-up and preservation approach.

We found differences in performance, meaning that after 30/90/180/365 days after the treatment concluded, we found that the likelihood of having a claim with a diagnosis of ulcer was reduced more using vCPM than other CTPs, which is a significant reduction of 36.7 percentage points in the case of vCPM versus other CTPs at 30 days after the conclusion of treatment. Performance equals reduction in the likelihood of having a claim with an ulcer diagnosis at the specified time period.

A study with similar methodology using Medicare claims also found that the AT performed better than no AT where performance measures were reductions in major and minor amputation, emergency department (ED) use, and hospital readmission.^20^ Patient selection issues were addressed in matching; however, there may be unobserved differences in the CTP populations.

An essential requirement to ensure the validity of a treatment effect estimated from an observational study is to adequately address confounding factor issues using control variables, such as patient demographic characteristics and comorbidities. Our study controlled for these variables and ensured that patients treated with different CTPs were matched. Even with strict controls on sample identification and episode creation, however, the treatment effect could only be associated with the outcome. Observational studies cannot assert causation of the outcome.

Skin substitutes are very frequently used by wound care physicians; however, due to an abundance of products in the market, evidence-based guidance is needed to help physicians in making an informed decision. The results reported analyze real-world evidence to help advance the knowledge for the broader technology of skin substitutes, where evidence is currently lacking.

In terms of policy, we found that CTPs are used in only a fraction of identified chronic DFUs. Expanded use of CTPs for DFUs may be a useful policy goal to improve the outcomes of DFU wound care. The findings of this study provide evidence that policymakers and payers might consider longer-term outcomes (6 month and 1 year) in addition to near-term process outcomes (such as wound closure rates), when making coverage determinations and setting payment policy.

### Limitations

This study applied Medicare administrative claims data. Information is, thus, limited to billed interactions between beneficiaries and qualifying providers. Patient selection issues were addressed through matching; however, there may be unobserved differences in the CTP populations that we were not able to account for.

Second, we assumed that all applications of CTP were applied to the same ulcer if the gap between two paid applications was no longer than 60 days because we could not definitively determine whether it was the same or a recurrent ulcer with the claims. Finally, the claims provide no information as to whether a wound closed, which, given its importance to treatment planning, is a significant limitation.

## Innovation

Enhanced coding specificity in observational claims studies improves identification of diabetic ulcers, especially by removing wounds with other potential etiologies (*e.g.*, venous, pressure). In clinical studies, treatment with vCPM has been shown to lead to higher wound closure rates as compared with devitalized placental tissue or bioengineered CTP with one cell type. Human placental membranes are a rich source of different cell types such as fibroblasts, epithelial cells, and stem cells along with collagen matrix and growth factors. Since different cells have different paracrine secretions, this may provide a benefit over other cellular and acellular CTPs.

**Key Findings**

- The vCPM CTP shows significant improvements over other cellular and acellular CTPs for reducing postepisode ulcer occurrence and mortality among Medicare beneficiaries receiving advanced wound care in the Medicare outpatient hospital setting.
- We believe this finding has major implications for patients with DFUs, and it may inform payers in setting coverage policies and access incentives that encourage providers to use vCPM CTPs with improved clinical outcome profiles.

## About the Authors

**Joan DaVanzo, PhD, MSW,** is Chief Executive Officer of Dobson DaVanzo and Associates, LLC. **Alex Hartzman, MS,** was a Senior Manager at Dobson DaVanzo and Associates, LLC when the article was written. He is now Director of Analytics at NORC at the University of Chicago. **Christopher Surfield, PhD,** was a Senior Manager at Dobson DaVanzo and Associates, LLC when the article was written. He is now an Economist at the Bureau of Economic Analysis. **Allen Dobson, PhD,** is President of Dobson DaVanzo and Associates, LLC.

## Author Confirmation

As submitting author, I attest that I have written consent from all authors to submit the article, and that all authors accept complete responsibility for the contents of the article. This article has not been previously published. It is not currently under consideration elsewhere. The work reported here will not be submitted for publication elsewhere until a final decision has been made as to its acceptability by *Advances in Wound Care*. Finally, I attest that the article is independent and original work, and all conclusions are from the authors. At the time of its writing, all the authors were employed by Dobson DaVanzo & Associates, LLC.

## Abbreviations and Acronyms

AT: advanced treatment
CCS: Condition Classification Software
CMS: Centers for Medicare & Medicaid Services
CONSORT: Consolidated Standards of Reporting Trials
vCPM: cryopreserved placental membrane containing viable cells
CTP: Cellular- and Tissue- Products
DFU: diabetic foot ulcer
ECM: extracellular matrix
FFS: Fee for Service
ICD-9-CM: International Classification of Disease, Ninth Revision, Clinical Modification
ICD-10-CM: International Classification of Disease, Tenth Revision, Clinical Modification
IPTW: inverse probability treatment weighting
LDS: Limited Data Set
